# The ACSL3-LPIAT1 signaling drives prostaglandin synthesis in non-small cell lung cancer

**DOI:** 10.1038/s41388-020-1196-5

**Published:** 2020-02-07

**Authors:** Maria Saliakoura, Inés Reynoso-Moreno, Chiara Pozzato, Matteo Rossi Sebastiano, Mirco Galié, Jürg Gertsch, Georgia Konstantinidou

**Affiliations:** 1 Institute of Pharmacology, University of Bern, 3010 Bern, Switzerland; 2 Institute of Biochemistry and Molecular Medicine, University of Bern, 3012 Bern, Switzerland; 3 Department of Neuroscience, Biomedicine and Movement, University of Verona, 37134 Verona, Italy

**Keywords:** Cancer metabolism, Phosphoinositol signalling

## Abstract

Enhanced prostaglandin production promotes the development and progression of cancer. Prostaglandins are generated from arachidonic acid (AA) by the action of cyclooxygenase (COX) isoenzymes. However, how cancer cells are able to maintain an elevated supply of AA for prostaglandin production remains unclear. Here, by using lung cancer cell lines and clinically relevant Kras^G12D^-driven mouse models, we show that the long-chain acyl-CoA synthetase (ACSL3) channels AA into phosphatidylinositols to provide the lysophosphatidylinositol-acyltransferase 1 (LPIAT1) with a pool of AA to sustain high prostaglandin synthesis. LPIAT1 knockdown suppresses proliferation and anchorage-independent growth of lung cancer cell lines, and hinders in vivo tumorigenesis. In primary human lung tumors, the expression of *LPIAT1* is elevated compared with healthy tissue, and predicts poor patient survival. This study uncovers the ACSL3-LPIAT1 axis as a requirement for the sustained prostaglandin synthesis in lung cancer with potential therapeutic value.

## Introduction

Arachidonic acid (AA) is a polyunsaturated fatty acid that, as arachidonate, is maintained at low concentrations, but it is highly abundant in its esterified form in membrane phospholipids. Therefore, the amount of AA is tightly controlled by the membrane phospholipid reacylation/deacylation cycle, known as the Lands cycle [1–3]. Depending on the cellular demand, AA can be released through phospholipid hydrolysis by phospholipase A2 (PLA2), phospholipase D, or phospholipase C pathways [4], and is then converted to prostaglandins by cyclooxygenases 1 and 2 (COX1 and COX2). Whereas COX1 is constitutively expressed, COX2 is induced by proinflammatory cytokines, and plays a central role in the insurgence of cancer inflammation and tumor progression [5, 6]. Notably, prostaglandins promote tumor growth both by directly activating signaling pathways, which control cancer cell proliferation, anchorage-independent growth, migration, and apoptosis, and by orchestrating interactions between tumor cells and the surrounding stromal cells, establishing an immunosuppressive tumor microenvironment [7–10].

Non-small cell lung cancer (NSCLC) constitutes about 85% of all lung malignancies, out of which 30% harbor KRAS mutations that are associated with aggressive, therapy-resistant tumors [11]. KRAS upregulates COX2, and the latter produces prostaglandins, including prostaglandin E2 (PGE2), in order to promote tumor growth and metastasis [12]. The transcriptional regulation of COX2 has been mainly attributed to MAPK signaling cascade, particularly to the ERK1/2, JNK/SAPK, and p38/RK/Mpk2 pathways [13–15]. However, it is unclear how KRAS is able to maintain a continuous supply of AA to feed COX2 and drive prostaglandin synthesis.

Mutant KRAS drives aberrant lipid metabolism in NSCLC by scavenging extracellular fatty acids [16, 17]. Indeed, we previously found that enhanced activity of the acyl-CoA synthetase long-chain 3 (ACSL3), an enzyme that catalyzes the activation of long-chain fatty acids to CoA thioesters, boosts extracellularly derived fatty acid activation in mutant KRAS NSCLC [17]. Knockdown of ACSL3 in NSCLC cell lines results in reduced cancer cell proliferation, while its deletion in mice suppresses Kras^G12D^-driven tumor initiation [17]. Thus, given the importance of ACSL3 in KRAS-driven tumorigenesis, and due to the fact that ACSL3 preferentially utilizes long-chain fatty acids, including arachidonate as substrates [18], we set out to investigate whether ACSL3 plays a role in mediating the KRAS-dependent prostaglandin production in lung cancer. To this aim, we combined mass spectrometry-based targeted lipidomics, in vivo cell-based assays, targeted genetic manipulations in cancer cells and in mouse models, as well as analysis of patient NSCLC samples.

## Results

### ACSL3 promotes channeling of AA to phosphatidylinositols in NSCLC cells

**Fig. 1:**
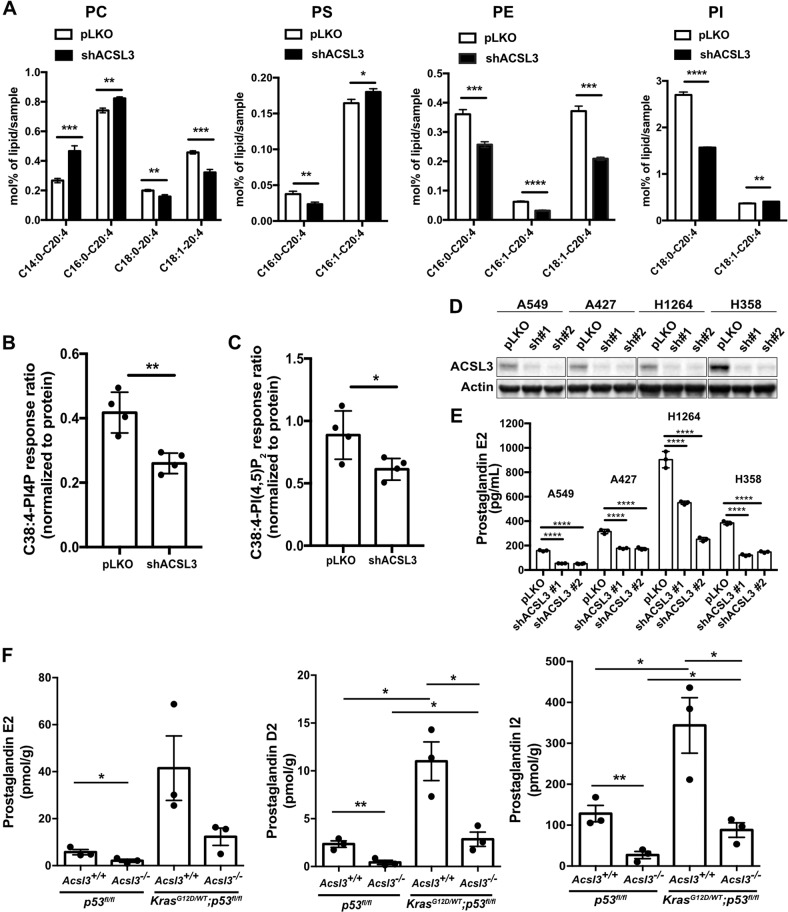
ACSL3 drives prostaglandin synthesis in NSCLC. **a** PC, PS, PE, and PI (mol%/sample) lipid alterations 72 h after ACSL3 knockdown in A549 cells. Cells were transduced with either an empty vector control (pLKO) or an shRNA against ACSL3 (shACSL3 #1), and 72 h later lipids were extracted and analyzed by mass spectrometry-based shotgun lipidomics. PC phosphatidylcholine, PS phosphatidylserine, PE phosphatidylethanolamine, PI phosphatidylinositol. *n* = 4/group. Data are presented as mean ± SD. C38:4-PI4P (**b**), C38:4-PI(4,5)P_2_ (**c**) peak areas (normalized to internal standard and protein content) after ACSL3 knockdown in A549 cells. Cells were transduced with either an empty vector control (pLKO) or an shRNA against ACSL3 (shACSL3 #1), and 72 h later lipids were extracted and analyzed by ultra-performance liquid chromatography-tandem mass spectrometry. *n* = 4/group. Data are presented as mean ± SD. **d** Immunoblot analysis of ACSL3 in A549 and A427, H1264, and H358 NSCLC cell lines transduced with either an empty vector control (pLKO) or two different shRNAs against ACSL3 and extracted 72 h later. **e** PGE2 ELISA assay for the indicated cell lines. Cells were transduced with either an empty vector control (pLKO) or two different shRNAs against ACSL3. PGE2 production was analyzed 24 h later. *n* = 3/group. Data are presented as mean ± SD. **f** Quantification of prostaglandin E2, D2, and I2 from *p53*^*flox/flox*^*;Acsl3*^*+/+*^ and *p53*^*flox/flox*^*;Acsl3*^*–/–*^ lungs, or from *Kras*^*G12D/WT*^*;p53*^*flox/flox*^*;Acsl3*^*+/+*^ and *Kras*^*G12D/WT*^*;p53*^*flox/flox*^*;Acsl3*^–*/*–^ macrodissected tumors, 10 weeks after tumor onset. Samples were extracted and analyzed by ultra-high-performance liquid chromatography with tandem mass spectrometry *n* = 3/group. Data are presented as mean ± SEM. Statistical analyses were done using two-tailed unpaired Student’s *t* test or one-way ANOVA. *p < 0.05, **p < 0.01, ***p < 0.001, ****p < 0.0001*.*

To understand whether ACSL3 is required to relay the KRAS-mediated AA cascade, we examined the lipid profile of the A549 NSCLC cell line upon ACSL3 knockdown by performing mass-spectrometry-based targeted lipidomics. We quantified the most abundant lipid species containing AA (20:4) in four phospholipid classes: phosphatidylcholine (PC), phosphatidylserine (PS), phosphatidylethanolamine (PE), and phosphatidylinositol (PI). We found that ACSL3 knockdown significantly altered the quantity of phospholipids containing AA in combination with 16:0 (palmitic), 16:1 (palmitoleate), 18:0 (stearate), and 18:1 (oleate) (Fig. 1a). Strikingly, the knockdown of ACSL3 resulted in a strong decrease in the AA content of PE and PI. Interestingly, we found a 40% decrease in PI containing C18:0–20:4 fatty acids (PI C18:0–20:4), the most abundant PI in mammalian cells [19–21] (Fig. 1a and Supplementary Fig. 1A). Of note, the knockdown of ACSL3 did not affect the protein levels of ACSL4, another ACSL with substrate preference for AA, underscoring the lack of compensatory effects (Supplementary Fig. 1B). These results strongly suggest that in mutant KRAS NSCLC cells, ACSL3 channels AA in glycerophospholipids.

PIs are reversibly phosphorylated at the inositol headgroup generating phosphoinositides, including phosphatidylinositol-4 phosphate (PI4P) and the plasma membrane-localized phosphatidylinositol-4, 5 biphosphate, PI(4,5)P_2_ [22, 23]. We hypothesized that a decrease in the most abundant PI, C18:0–20:4, would also affect the production of C18:0–20:4 PI4P and C18:0–20:4 PI(4,5)P_2_. Indeed, upon ACSL3 knockdown, we observed a decrease in C38:4–PI4P and C38:4–PI(4,5)P_2_ lipids presumably due to the reduction of PI C18:0–C20:4 (Fig. 1b, c). This result additionally confirms a role of ACSL3 in esterifying AA into PI in mutant KRAS lung cancer cells.

### ACSL3 drives prostaglandin synthesis in NSCLC

PI is the major source of AA, and in our lipidomic analysis, the AA-containing PI was consistently downregulated upon ACSL3 knockdown (Fig. 1a). Thus, we hypothesized that reduced AA-containing PIs would result in reduced prostaglandin synthesis. To this aim, we quantified PGE2, a prostaglandin produced by PGE2 synthase from COX-derived prostaglandin H2 [24]. We found that PGE2 was strongly reduced upon ACSL3 knockdown in a panel of NSCLC cell lines harboring KRAS mutations, namely A549, A427, H1264, and H358, and this coincided with decreased cell proliferation (Fig. 1d, e and Supplementary Fig. 1C). To investigate whether ACSL3 knockdown impairs PGE2 production in lung cancer cells carrying wild-type KRAS, we assessed four representative cancer cell lines with known differential sensitivity to ACSL3 knockdown, namely H596, H838, H125, and HCC95 (ref. [17] and Supplementary Table 1). The proliferation of H596 and H838 cell lines was unaffected by ACSL3 knockdown (hereafter these cells are mentioned as ACSL3-independent), while the proliferation of H125 and HCC95 is significantly reduced (hereafter these cells are mentioned as ACSL3-dependent) (Supplementary Figs. 1D and 1E). We measured PGE2 upon ACSL3 knockdown with two different shRNAs, and found that the loss of ACSL3 caused either a mild decrease (H596) or had no effect (H838) in PGE2 production in the ACSL3-independent cancer cell lines, while it highly suppressed PGE2 production in the ACSL3-dependent cell lines H125 and HCC95 (Supplementary Fig. 1F).

To confirm these results in vivo, we generated a mouse model bearing a Cre-activatable mutant Kras allele (*LSL-Kras*^*G12D/WT*^*)*, homozygous for a Cre-conditional *p53-*knockout allele (*p53*^*flox/flox*^) [25, 26] and either wild type (*LSL-Kras*^*G12D/WT*^*;p53*^*flox/flox*^*;Acsl3*^*+/+*^) or knockout for *Acsl3* (*LSL-Kras*^*G12D/WT*^*;p53*^*flox/flox*^*;Acsl3*^–*/*–^). In the *LSL-Kras*^*G12D/WT*^*;p53*^*flox/flox*^ model, Cre-mediated loss of a stop cassette permits expression of the oncogenic *Kras*^*G12D*^ allele from its endogenous promoter, and recapitulates key features of the human disease, including histologic features and response to conventional and targeted therapies [27]. Of note, we have previously shown that the *LSL-Kras*^*G12D/WT*^*;Acsl3*^–*/*–^ mice display impaired lung tumor initiation and progression compared with their wild-type littermates [17]. Therefore, we employed this mouse model to assess prostaglandin production in mouse lungs 10 weeks after adenoviral-mediated Cre delivery to the lungs. In both genotypes, the level of different prostaglandins (PGE2, PGD2, and PGI2) was significantly increased in tumor lesions compared with healthy lungs (Fig. 1f). However, we found in both lung tumors and healthy lungs that Acsl3 knockout causes a striking reduction of the aforementioned prostaglandins (Fig. 1f). Thus, our results suggest that ACSL3 is important for prostaglandin synthesis regardless of the oncogenic mutational status. Nevertheless, our data indicate that ACSL3 significantly enhances prostaglandin synthesis in tumors compared with healthy lung, suggesting that this may fulfill the increased requirement for prostaglandin synthesis of lung cancer.

### LPIAT1 mediates prostaglandin production and promotes the proliferation and anchorage-independent growth of NSCLC cells

**Fig. 2:**
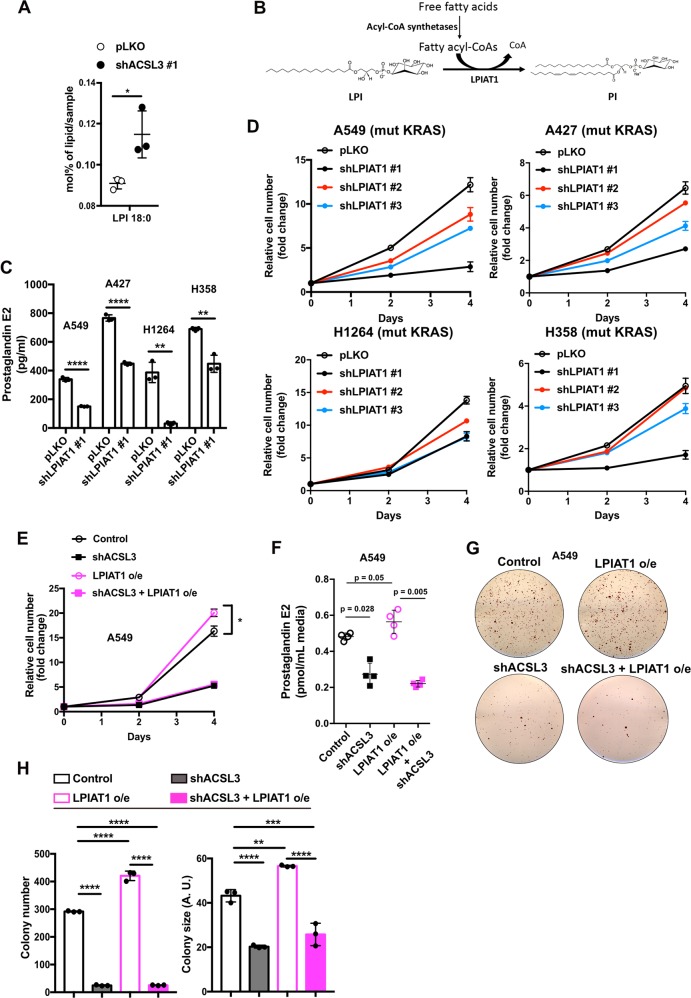
LPIAT1 requires ACSL3-derived arachidonoyl-CoA for prostaglandin synthesis. **a** Lysophosphatidylinositol (LPI) 72 h after ACSL3 knockdown in A549 cells. Cells were transduced with either an empty vector control (pLKO) or an shRNA against ACSL3 (#1), 72 h later lipids were extracted and analyzed by mass spectrometry-based shotgun lipidomics *n* = 4/group. Data are presented as mean ± SD. **b** Schematic model for LPIAT1 function in phospholipid remodeling. **c** PGE2 ELISA assay for A549, A427, H1264, and H358 cell lines. Cells were transduced with either an empty vector control (pLKO) or shRNA against LPIAT1 (shLPIAT1 #1). Cells were transduced, selected with puromycin and plated for PGE2 measurement. 24 h later the media was changed, incubated for additional 24 h and PGE2 production was quantified from the supernatant *n* = 3/group. Data are presented as mean ± SD. **d** Relative cell number of A549, A427, H1264, and H358 cell lines transduced with an empty vector control (pLKO) or three different shRNAs against LPIAT1. *n* = 3/group. Data are presented as mean ± SD. **e** Relative cell number of A549 cell line transduced with either pLKO.1 hygro + pLenti-GIII-CMV-GFP-2A-Puro (control) or pLKO.1 hygro-shACSL3 #2 + pLenti-GIII-CMV-GFP-2A-Puro (shACSL3) or pLKO.1 hygro + pLenti-GIII-CMV-GFP-2A-Puro-LPIAT1 (LPIAT1 overexpression) or pLKO.1 hygro-shACSL3 #2 + pLenti-GIII-CMV-GFP-2A-Puro-LPIAT1 (shACSL3 + LPIAT1 overexpression) *n* = 3/group; o/e: overexpression. Data are presented as mean ± SD. **f** PGE2 quantification from A549 cell line supernatants. Cells were transduced with either pLKO.1 hygro + pLenti-GIII-CMV-GFP-2A-Puro (control) or pLKO.1 hygro-shACSL3 #2 + pLenti-GIII-CMV-GFP-2A-Puro (shACSL3) or pLKO.1 hygro + pLenti-GIII-CMV-GFP-2A-Puro-LPIAT1 (LPIAT1 overexpression) or pLKO.1 hygro-shACSL3 #2 + pLenti-GIII-CMV-GFP-2A-Puro-LPIAT1 (shACSL3 + LPIAT1 overexpression). Cells were transduced, selected with 2 μg/mL puromycin or 150 ng/mL hygromycin and plated for PGE2 measurement. 24 h later the media was changed, incubated for additional 24 h and Supernatants were analyzed by ultra-high performance liquid chromatography with tandem mass spectrometry *n* = 4/group. Data are presented as mean ± SD. **g** Representative images of soft agar colony formation assay in A549 cells transduced as in **f**. **h** Histograms showing quantification of colony number and size of A549 cells grown in soft agar treated as in **f**. *n* = 3/group. AU arbitrary units. Data are presented as mean ± SD. Statistical analyses were done using two-tailed unpaired Student’s *t* test or one-way ANOVA. *p *<* 0.05, **p *<* 0.01, ****p *<* 0.0001.

Glycerophospholipids are remodeled by the action of phospholipases that hydrolyze them at the *sn-2* position to yield a lysophospholipid and a free fatty acid, while their reacylation is catalyzed by lysophospholipid acyltransferases [28, 29]. Data from our lipidome profiling show that ACSL3 knockdown in A549 cells led to a reduction in C18:0–C20:4 PI, which could be caused by a decrease in C18:0-lysophosphatidylinositol (LPI) to C18:0–C20:4 PI production (Fig. 1a). Indeed, we found an accumulation of C18:0-LPI, suggesting that ACSL3 knockdown causes a block of LPI–PI conversion by reducing the supply of arachidonoyl-CoA (Fig. 2a).

The enzyme responsible for the conversion of LPI–PIs with specificity for arachidonyl-CoA is the lysophosphatidylinositol-acyltransferase-1 (LPIAT1) (Fig. 2b) [30]. Thus, we hypothesized that LPIAT1 may be an important mediator of prostaglandin signaling downstream of ACSL3. Indeed, RNA interference-mediated LPIAT1 knockdown led to a significant decrease in PGE2 production in the mutant KRAS NSCLC cell lines, A549, A427, H1264, and H358 (Fig. 2c and Supplementary Fig. 2A). Next, we investigated whether LPIAT1 knockdown suppresses PGE2 production in wild-type KRAS NSCLC cell lines. LPIAT1 knockdown caused either a mild decrease (H596) or had no effect (H838) in PGE2 production in ACSL3-independent cancer cell lines, while significantly decreased PGE2 production in the ACSL3-dependent cell lines, H125 and HCC95 (Supplementary Figs. 2B and 2C). Moreover, in order to understand the impact of LPIAT1 depletion on cancer cell proliferation, we knocked down LPIAT1 in the aforementioned NSCLC cell lines. We found that LPIAT1 significantly suppressed the proliferation of mutant KRAS NSCLC cell lines A549, A427, H1264, and H358 (Fig. 2d) as well as that of ACSL3-dependent wild-type KRAS NSCLC cell lines, H125 and HCC95 but had no impact on the proliferation of H596 and H838 cell lines (Supplementary Fig. 2D). These results suggest that the ACSL3/LPIAT1 metabolic signaling axis plays an important role in prostaglandin production and cell proliferation of NSCLC cells.

To assess whether LPIAT1 requires ACSL3-derived substrates to control cancer cell proliferation, we overexpressed LPIAT1 with a lentiviral construct in A549 and H358 cell lines and performed cell proliferation assays upon ACSL3 knockdown. Interestingly, although the overexpression of LPIAT1 enhanced cancer cell proliferation, this effect was completely abolished upon ACSL3 knockdown, thus strongly suggesting that LPIAT1 requires ACSL3-derived arachidonyl-CoA to promote cancer cell proliferation (Fig. 2e and Supplementary Fig. 2E, 2F). Accordingly, measurement of PGE2 in A549 cells evidenced that in the absence of ACSL3, PGE2 production is compromised even when LPIAT1 is overexpressed (Fig. 2f). Next, we performed soft agar colony formation assays and we found that LPIAT1 overexpression increased the number and size of colonies, while the combination with ACSL3 knockdown abolished this effect (Fig. 2g, h). These data suggest that LPIAT1 requires ACSL3 to control cell proliferation and anchorage-independent growth in NSCLC cells.

### LPIAT1 knockdown suppresses tumorigenesis and improves the survival of mice bearing human lung cancer xenografts

**Fig. 3:**
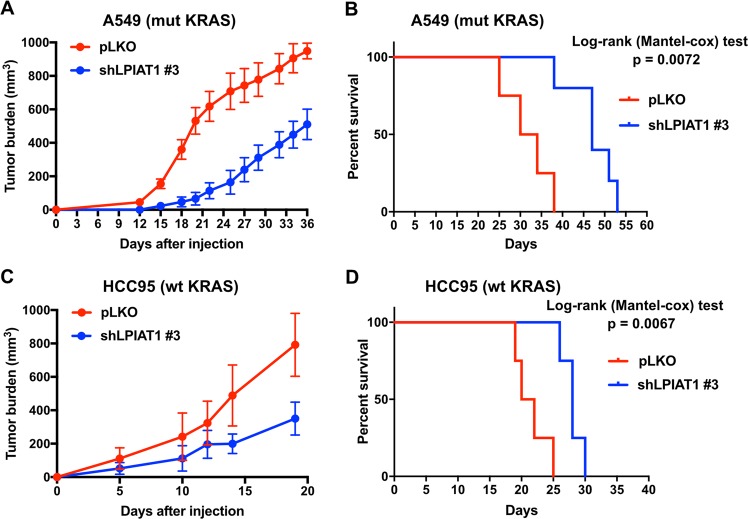
LPIAT1 knockdown reduces tumorigenesis and extends survival of mice. **a, c** Tumor burden of A549 (mut KRAS) and HCC95 (wt KRAS) cells grown as xenografts in immunocompromised (NSG) mice. A549 or HCC95 cells were transduced with either a control (pLKO empty vector) or an shRNA against LPIAT1 (shLPIAT1 #3) and 1 × 10^6^ A549 or 2.5 × 10^6^ HCC95 cells were injected subcutaneously in mice. Tumors were measured every 2–3 days with a caliper. Data in **a** are presented as mean ± SEM and in **c** as ± SD. **b**, **d** Kaplan–Meier survival curve of mice from A549 (**b**) or HCC95 (**d**) from the experiment shown in **a** and **c**, respectively. Statistical analyses were done using log-rank (Mantel-cox) test. A549 pLKO *n* = 4, A549 shLPIAT1 *n* = 5, HCC95 pLKO *n* = 4, HCC95 shLPIAT1 *n* = 4.

Our results suggest that LPIAT1 may be essential for KRAS-mediated prostaglandin production in lung cancer cells. In order to expand the significance of our finding in vivo, we injected the A549 (mutant KRAS) and HCC95 (wild-type KRAS, ACSL3-dependent) NSCLC cells into immunocompromised NOD scid gamma (NSG) mice, previously transduced with a control or shRNA against LPIAT1. In this model, we found that knockdown of LPIAT1 significantly suppressed tumorigenesis in mice and improved their overall survival (Fig. 3a–d). These results indicate that LPIAT1 plays a role in tumor progression.

### LPIAT1 is upregulated in human NSCLC and positively correlates with poor patient survival

**Fig. 4:**
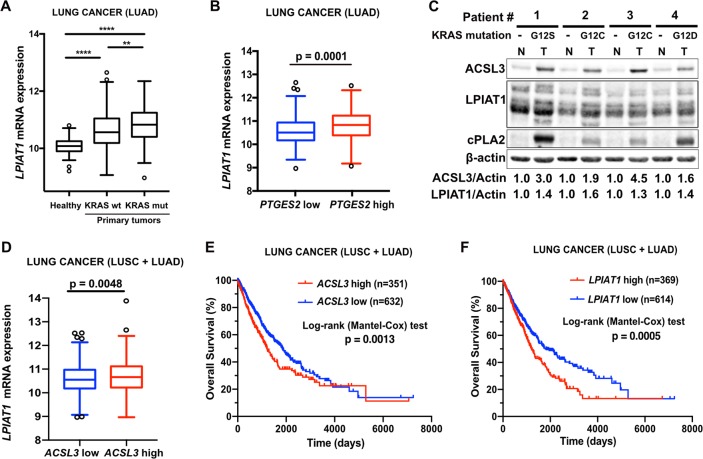
*LPIAT1* is overexpressed in human lung cancer and predicts poor patient survival. **a** Relative *LPIAT1* mRNA expression in healthy lungs (*n* = 59) or lung tumors from wild-type (*n* = 326) or mutant KRAS (*n* = 148) human lung adenocarcinomas (LUAD cohort). The box-plots span from the first to third quartile (depicting the median as a line in the middle), the whiskers extend to 1.5 x IQR (interquartile range). Outliers > 1.5 times the IQR are indicated with circles. **b** Relative *LPIAT1* mRNA expression in LUAD cohort stratified by *PTGES2*-high (*n* = 258) versus *PTGES2*-low (*n* = 259) by median separation. Outliers >1.5 times the IQR are indicated with circles. **c** Immunoblot analysis of the indicated targets in primary human patient-derived lung adenocarcinoma samples. N normal lung, T lung tumor (lung adenocarcinomas). The mutational status of KRAS is indicated. **d** Relative *LPIAT1* mRNA expression in squamous lung carcinoma (LUSC) and lung adenocarcinoma (LUAD) cohorts stratified by *ACSL3*-high (*n* = 508) versus *ACSL3*-low (*n* = 509) by median separation. Outliers > 1.5 times the IQR are indicated with circles. Kaplan–Meier analysis showing the percent survival of *ACSL3*-low versus *ACSL3*-high (**e**) or *LPIAT1*-low versus *LPIAT1*-high (**f**) in squamous lung carcinoma (LUSC) and lung adenocarcinoma (LUAD) cohorts. The number of patients is indicated. Statistical analyses were done using two-tailed unpaired Student’s *t* test, one-way ANOVA or log-rank (Mantel-cox) test. **p < 0.01, ****p < 0.0001*.*

To explore the relevance of *LPIAT1* in lung cancer, we investigated a lung adenocarcinoma cohort (subset LUAD that includes information on KRAS mutational status) from the The Cancer Genome Atlas (TCGA) database, to compare the gene expression of *LPIAT1* between wild-type KRAS tumors, mutant KRAS tumors and healthy lung tissue [31]. Our analysis evidenced a higher *LPIAT1* expression in lung tumors compared with healthy lung tissue samples (Fig. 4a). However, the expression of *LPIAT1* was higher in tumors with *KRAS* mutations compared with tumors carrying wild-type *KRAS* allele (Fig. 4a). Moreover, high *LPIAT1* expression highly correlated with high *Prostaglandin E Synthase* expression, an enzyme that catalyzes the conversion of prostaglandin H2 to PGE2 (Fig. 4b). These data suggest that high *LPIAT1* expression is not restricted to mutant *KRAS* tumors and underscore a broader relevance of *LPIAT1* in NSCLC.

To validate the database analysis, we performed immunoblot using human patient-derived NSCLC biopsies with known KRAS mutations and the corresponding adjacent healthy tissue. Our results indicated that the protein levels of ACSL3, LPIAT1 and the cytosolic phospholipase A2 (cPLA2), an enzyme that catalyzes the hydrolysis of membrane phospholipids to release AA for prostaglandin and other eicosanoid production, are upregulated in patient-derived mutant KRAS tumors compared with adjacent healthy tissue (Fig. 4c). Thus, these data confirm that the ACSL3-LPIAT1 metabolic pathway is enhanced in NSCLC.

To assess the relationship between *ACSL3* and *LPIAT1* expression, we employed a NSCLC cohort that includes squamous lung carcinomas (LUSC) and lung adenocarcinomas (LUAD), stratified by *ACSL3*-high and *ACSL3*-low expression, and we found a direct correlation between the expression levels of *LPIAT1* and *ACSL3*, suggesting a co-regulation of these enzymes in NSCLC (Fig. 4d).

Next, we examined the relationship between patient survival and *ACSL3* or *LPIAT1* expression. Kaplan–Meier analysis of LUSC and LUAD patient cohorts stratified by high versus low *ACSL3* or *LPIAT1*, evidenced that patients with either high *ACSL3* or high *LPIAT1* expression had lower overall survival (Fig. 4e, f). These results suggest that both *ACSL3* and *LPIAT1* overexpression are clinically relevant and may have prognostic value for survival outcomes in NSCLC patients.

## Discussion

Elevated prostaglandin levels have been extensively associated with enhancement of cancer cell survival and tumor growth, migration, invasion, and immunosuppression [3]. In several types of cancer, including mutant KRAS lung tumors, an important part of this effect has been attributed to the enhanced activity of COX1 and 2, the enzymes responsible for the production of prostaglandins from AA [32–34]. However, how the metabolism of AA is remodeled in cancer cells to cope with the high demand for prostaglandin synthesis remains elusive. Here, we found that, in mutant KRAS and in a subset of wild-type KRAS lung cancer cells, high prostaglandin levels are sustained by LPIAT1 activity and depend on the ACSL3-activated AA substrate availability (Fig. 2 and Supplementary Fig. 2). Importantly, the ACSL3-LPIAT1 metabolic axis drives prostaglandin synthesis to promote tumorigenesis in NSCLC (Fig. 3). We found that a subset of wild-type KRAS cancer cells show virtually no effect in PGE2 suppression and cell proliferation upon ACSL3 or LPIAT1 knockdown. These data suggest that alternative signaling pathways may confer resistance to ACSL3 or LPIAT1 inhibition and future studies will be necessary to identify these mechanisms. For instance, since the production of free AA is highly regulated by a PLA2-dependent deacylation reaction and a LPIAT1-dependent reacylation/transfer into PI pools, PLA2 overexpression may result in increased release of free AA available for prostaglandin synthesis leading to resistance to LPIAT1 inhibition. Indeed, cPLA2 overexpression is common in NSCLC [35]. Moreover, PLA2 enzymes can also liberate free AA from other phospholipids such as PC and PS [36]. Importantly, we found an upregulation of some PC and PS species bound to AA (C14:0–C20:4 PC, C16:0–C20:4 PC, and C16:1–C20:4 PS) upon ACSL3 knockdown, suggesting that ACSL3 suppression results in a remodeling of PC and PS membrane phospholipids that PLA2 could, in principle, deacylate to provide free AA for prostaglandin synthesis (Fig. 1a).

Our data show that cancer cell lines that are sensitive to ACSL3 knockdown are also sensitive to LPIAT1 knockdown, while others that are resistant to ACSL3 knockdown are also resistant to LPIAT1 knockdown (Supplementary Fig. 1D-1F and Supplementary Fig. 2B–2D). We also found that overexpression of LPIAT1 causes enhanced cancer cell proliferation and increased colony formation, an effect that was rescued by knocking down ACSL3 (Fig. 2e, h). In this context, knockdown of ACSL3 reduced the availability of arachidonoyl-CoA, the substrate of LPIAT1, hence reducing the most abundant PI species (C18:0–20:4 PI) and prostaglandin synthesis (Figs. 1a, d and 2f). Thus, our data indicate that ACSL3 and LPIAT1 regulate cancer cell proliferation, at least in part, by acting on the same metabolic axis. Whether the overexpression of ACSL3 is sufficient to increase synthesis of prostaglandins and promote cancer proliferation remains to be investigated.

We previously found that ACSL3 is required for fatty acid β-oxidation in NSCLC cells [17]. Therefore, it is plausible that LPIAT1 and ACSL3 can sustain lung cancer proliferation by also serving other pathways. In this regard, it has been recently shown that during human platelet activation, prostaglandins and other eicosanoids are fed at high rates into β-oxidation in a cPLA2-dependent manner. Accordingly, cPLA2 blockade decreased β-oxidation and impaired mitochondrial respiration [37]. Thus, it would be of interest to investigate whether lung cancer cells rely on prostaglandins as substrates to support β-oxidation.

Although nonsteroidal anti-inflammatory drugs, which target COX enzymes and specific COX2 inhibitors, are among the most promising drugs against cancer, the serious cardiovascular and gastrointestinal side effects have reduced the enthusiasm for their use [38]. Therefore, the identification of novel tumor-specific targets related to AA remodeling may help develop therapies with a greater benefit and fewer side effects. Our results suggested that prostaglandin synthesis is higher in lung tumors compared with healthy tissue (Fig. 1f). Of note, mice with germline deletion of *Acsl3* are viable and do not show any overt dysfunctions [17]. Thus, in principle, pharmacologic targeting of the ACSL3-LPIAT1 axis may benefit patients to selectively target tumor-derived prostaglandin synthesis while sparing normal cellular functions. However, germline deletion of LPIAT1 in mice results in defective brain development and mortality [39]. It would therefore be of interest to determine whether post-developmental knockout or inhibition of LPIAT1 would lead to a similar or other body dysfunctions, and whether it could be used for cancer treatment in the future. Notably, we found that high *LPIAT1* expression predicts poor survival in mouse xenografts and human NSCLC patients (Figs. 3, 4). Hence, the status of ACSL3-LPIAT1 axis in human lung tumors may serve as a biomarker for personalized anti-cancer treatment.

Increased synthesis of prostaglandins is a negative prognostic marker in lung cancer and several other malignancies (e.g., gastric, colorectal, breast, hepatic, bladder, and renal cancers) [10, 40, 41]. Thus, future experiments aimed at assessing the role of LPIAT1 in prostaglandin synthesis and tumorigenesis in other types of cancer are warranted.

In conclusion, we have unraveled the ACSL3-LPIAT1 metabolic axis as a requirement for prostaglandin production and tumorigenesis in NSCLC that could be exploited for therapeutic intervention.

## Materials and methods

### Resource sharing

Further information and requests for resources and reagents should be directed to, and will be fulfilled by the Lead Contact, Georgia Konstantinidou, Institute of Pharmacology, University of Bern, 3010 Bern, Switzerland (georgia.konstantinidou@pki.unibe.ch). Requests will be handled according to the University of Bern policies regarding MTA and related matters.

### Reagents and plasmids

pLKO.1 puro (Addgene plasmid #8453; http://n2t.net/addgene:8453; RRID:Addgene_8453), pLKO.1 hygro (Addgene plasmid #24150; http://n2t.net/addgene:24150; RRID:Addgene_24150), pCMV-VSV-G (Addgene plasmid #8454; http://n2t.net/addgene:8454; RRID:Addgene_8454), and pCMV-dR8.2 dvpr (Addgene plasmid #8455; http://n2t.net/addgene:8455; RRID:Addgene_8455) were a gift from Prof. Bob Weinberg [42]. The human LPIAT1-containing lentiviral vector (pLenti-GIII-CMV-GFP-2A-Puro) was purchased from Applied Biological Materials Inc. The ACSL3 and LPIAT1 shRNAs were obtained as bacterial glycerol stock from Sigma-Aldrich and the sequence of interest was subcloned into the pLKO-puro backbone or into pLKO-hygro (for the combination studies with LPIAT1 overexpression) plasmids after digestion with AgeI/EcoRI. The final shRNA constructs were confirmed with sequencing.

### Cell lines

All human NSCLC cell lines used in this study (A549, H358, H1264, A427, H838, H596, H125, and HCC95) were derived from male patients, and were provided by Dr. John Minna (UT Southwestern Medical Center) [43]. All cell lines were DNA fingerprinted for provenance. Cell lines were screened free for mycoplasma, and cultured in an incubator at 37 ^o^C and 5% CO_2_ in RPMI-1640 medium (Gibco) containing 10% fetal bovine serum (Thermo Fisher), 100 I.U./mL penicillin, 100 μg/mL streptomycin, and 0.5 μg/mL puromycin (Gibco).

### Animal studies

Mice were maintained under controlled humidity and temperature conditions, with a standard 12-h light/dark cycle and were fed ad libitum. Mixed background *LSL-Kras*^*G12D/WT*^*;p53*^*flox/flox*^ mice were generated by crossing stock *B6.129SS4-kras*^*tm4Tyj*^*/J* (from JaxLab, Stock number 008179) [25], with *B6.129P2-Trp53*^*tm1Brn*^*/J* (from JaxLab, Stock number 008462) mouse [26]. The mixed background Cre-inducible *LSL-Kras*^*G12D/WT*^*;p53*^*flox/flox*^;*Acsl3*^–*/*–^ mouse model was obtained by crossing *LSL-Kras*^*G12D/WT*^*;p53*^*flox/flox*^ mice with B6;129S5-Acsl3^*Gt(OST148301)Lex*^*/*Orl [44]. *LSL-Kras*^*G12D/WT*^*;p53*^*flox/flox*^*;Acsl3*^*-/-*^ mice were backcrossed for eight generations, before creating the experimental groups. The NOD.Cg-Prkdc^scid^ Il2rg^tm1Wjl^/SzJ NSG mice were from Jackson labs (stock number: 005557). Only male littermates were used for the experiments. Animal handling and experimental procedures were performed in compliance with the federal guidelines and were approved by the Veterinaerdienst de Kantons Bern.

For intratracheal injections, 2.5 × 10^7^ infectious particles of VVC-U of Iowa-5 Ad5CMVCre (Viral Vector Core, University of Iowa) were delivered to male mice at 8 weeks of age, resulting in the concomitant lung specific *Kras*^*G12D/WT*^ induction, and *p53* deletion. Mice were sacrificed 10 weeks post induction, and lungs were retrieved after anesthesia and perfusion of the animal with 20 ml of PBS. Healthy lung tissue and macrodissected tumors used for measurement of prostaglandin levels were snap-frozen in liquid nitrogen.

For the xenotransplantation study in vivo, 1 × 10^6^ A549 cells (A549 pLKO or A549 shLPIAT1 #3) or 2.5 × 10^6^ HCC95 cells (HCC95 pLKO or HCC95 shLPIAT1 #3) resuspended in 100 μl of sterile PBS were injected subcutaneously to male NOD.Cg-Prkdc^scid^ Il2rg^tm1Wjl^/SzJ NSG mice at 7–8 weeks of age. The mice were closely monitored on a daily basis, and the size of the tumors was measured with a caliper every 2–3 days. Mice were sacrificed when the tumor volume reached 1000 mm^3^.

Genomic DNA extraction and PCR assay were performed using the KAPA HotStart Mouse Genotyping Kit (Kapa Biosystems, KK7352) and KAPA2G Fast HotStart Genotyping Mix (Kapa Biosystems, KK5621), respectively, according to the manufacturer’s instructions. The mice genotypes were confirmed following the corresponding provider’s protocols. The full list of oligos used to genotype the mice can be found in Supplementary Table 2.

### Human studies

The patient-derived frozen lung adenocarcinoma samples used for Fig. 4 were provided by the institute of pathology, translational research unit. The sex of the patients is the following: Patient #1: female, #2: female, #3: male, and #4: female. The use of human samples was approved by the ethics commission (swissethics), ID: 2017-01322. All samples were provided upon patients' consent.

### TCGA data analysis

TCGA LUSC and lung adenocarcinoma (datasets LUSC and LUAD, respectively) datasets were retrieved from http://cancergenome.nih.gov. The data were downloaded with the help of the web graphic user interface Xenabrowser (xenabrowser.net) [45] and analyzed with GraphPad Prism version 7.0. Incomplete data, missing expression values and/or survival were eliminated from the analysis and only primary tumors were considered. RSEM gene quantifications as provided by TCGA were taken. The stratification in high-/low-expressing groups in Fig. 4b, d was performed by median separation as indicated in the related figure legends. For the patient survival analysis (Fig. 4e, f), patients were classified into two groups, and association between prognosis (survival) and gene expression (FPKM) was examined. The best expression cut-off refers the FPKM value that yields maximal difference with regards to survival between the two groups at the lowest log-rank *P* value.

### shRNAs, virus production, and transduction

Recombinant lentiviruses were produced by transfecting HEK 293 T cells, using the *Trans*IT^®^-293 Transfection Reagent (Mirus; MIR2705), with pCMV-VSV-G (VSV-G protein), pCMV-dR8.2 (lentivirus packaging vector), and lentiviral constructs, according to the manufacturer’s instructions.

### Mass spectrometry-based shotgun lipidomics

#### Lipid extraction

Mass spectrometry-based lipid analysis was performed by Lipotype GmbH (Dresden, Germany) as previously described [46]. Lipids were extracted using a two-step chloroform/methanol procedure [47]. Data analysis and post-processing. Data were analyzed with in-house developed lipid identification software based on LipidXplorer [48, 49]. Data post-processing and normalization were performed using an in-house developed data management system. Only lipid identifications with a signal-to-noise ratio >5, and a signal intensity fivefold higher than that in the corresponding blank samples, were considered for further data analysis. Experimenters were blinded during data analysis.

### Ultra-performance liquid chromatography-tandem mass spectrometry

#### Reagents

Methanol, chloroform, dichloromethane, and acetonitrile (Fisher) were all of mass spectrometry grade. Sodium formate and HCl were from Sigma, and TMS-diazomethane (TMS-DM, 2.0 M in hexanes) from Sigma-Aldrich and Acros. Lipid standards were ammonium salts of 1-heptadecanoyl-2-(5Z,8Z,11Z,14Z-eicosatetraenoyl)-sn-glycero-3-phospho-(1′-myo-inositol-4′,5′-bisphosphate) [17:0-20:4 PI(4,5)P_2_] Avanti Polar Lipids, LM1904; 1-heptadecanoyl-2-(5Z,8Z,11Z,14Z-eicosatetraenoyl)-sn-glycero-3-phospho-(1′-myo-inositol-4′-phosphate) [17:0-20:4 PI(4)P] Avanti Polar Lipids, LM1901; and 1-heptadecanoyl-2-(5Z,8Z,11Z,14Z-eicosatetraenoyl)-sn-glycero-3-phospho-(1′-myo-inositol-3′,4′,5′-trisphosphate) [17:0-20:4 PI(3,4,5)P_3_] Avanti Polar Lipids, LM1906, Trimyristin (14:0, 14:0, 14:0), Tripalmitin (16:0, 16:0, 16:0), Triolein (18:1, 18:1, 18:1) Trilinolein (18:2,18:2,18:2), Tristearin (18:0,18:0,18:0), Triarachidin (20:0,20:0,20:0), Triarachidonin (20:4,20:4,20:4) (NuChek-Prep, Inc. Elysian, MN).

#### Sample processing

Cells (2 × 10^6^) were washed twice with PBS and incubated with 0.5 M trichloroacetic acid (TCA) for 5 min on ice. Cells were then scraped from the dish, vortexed for 30 s and further incubated on ice for 5 min. The TCA-treated samples were centrifuged at 20,000 × *g* for 3 min at 4 ^o^C. After discarding the supernatant, the pellet was resuspended in 1 mL of 5% (w/v) TCA + 10 mM EDTA and centrifuged at 20,000 × *g* for 3 min at 4 ^o^C. After repeating the same step once, the pellet was used for the lipid extraction.

#### Lipid extraction

Prior to lipid extraction the following lipid analytical internal standards were added to the TCA precipitates: 17:0–20:4 PI(4,5)P_2_, 17:0–20:4 PI(3,4,5)P_3_, 17:0–20:4 PIP. Lipids were extracted using a modification of an acidified chloroform-methanol extraction protocol [50, 51]. It was initiated by adding 670 µL of chloroform:methanol:12 N HCl (40:80:1) to the TCA precipitate followed by vigorous vortexing for 5 min and incubation for 10 min at 4 °C. Then 650 µL of ice-cold chloroform was added and the samples were vortexed for another 2 min and allowed to sit for 5 min at 4 °C after which 300 µL of ice cold 1 M HCl was added. The samples were vortexed for 2 min, centrifuged at 10,000 × *g* for 2 min, and the lower phase was then collected in a fresh 2-mL microcentrifuge tube. Ice cold theoretical lower phase (900 μl) generated by combining chloroform:methanol:1.74 M HCl mixture (86:14:1,v/v/v) was added to the upper phase and the mixture was vortexed and centrifuged. The lower phase was then combined with the previously collected lower phase and dried under a stream of N_2_ and subsequently methylated as previously described [52].

#### Mass spectroscopy

LC/MS was carried out essentially as previously described [52]. Aliquots of sample resuspended in 20–100 µL of 100% mass spectroscopy grade methanol were injected with a Waters Acquity FTN autosampler into the UPLC/MS. Chromatography over a Waters Acquity UPLC C4 column (Waters Acquity UPLC Protein BEH C4, 1.7 μm 1.1 × 100; 300 A) was carried out with an acetonitrile formic acid gradient monitored by a Waters XEVO TQ-S MS/MS in multiple reaction monitoring mode using electrospray and positive ion mode. The gradient was initiated with 10 mM formic acid in water/10 mM formic acid in acetonitrile (33:67 v/v), held for 1 min, then increased to 15:85, v/v in 9 min following injection, held at 85% for 1 min, then increased to 100% for an additional 3 min, and then re-equilibrated to starting conditions for 3 min.

### Quantification of PGE2, PGD2, and PGI2 by LC–MS/MS

#### Sample preparation

Lung tissue samples were weighted while still frozen and transferred into 2 mL XXTuff reinforced microvials (Bio Spec Products Inc., OK, USA) with three chrome-steel beads (diameter, 2.3 mm; Bio Spec Products Inc., OK, USA) and the corresponding volume of 0.1 M formic acid to reach 100 mg/mL. Samples were homogenized using a Mini-Beadbeater-24 (Bio Spec Products Inc., OK, USA) and the extraction of AA, PGE2, PGD2, and PGI2 (since PGI2 is not metabolically stable, this analyte was quantified using its stable hydrolysis product 6k-PGF1a) as previously described [53]. LC–MS/MS conditions. LC–MS/MS analysis was performed using our previously described protocol [53] with some changes. We used a Shimadzu UFLC coupled to a TripleQuad 5500 QTRAP mass spectrometer (AB Sciex, Canada). The LC column was a Reprosil-PUR C18 column (3 μm particle size; 2 × 50 mm; Dr. A. Maisch HPLC GmbH, Germany) maintained at 40 °C with a mobile phase flow rate of 0.3 mL/min. The mobile phase composition was a mixture of (A) 2 mM ammonium acetate plus 0.1% formic acid and (B) acetonitrile plus 0.1% formic acid. A gradient elution was used, starting with 95% of phase A and linear increase of phase B reaching 40% at minute 3; then the linear increase rate was decreased to reach 65% B at minute 9. Finally, to flush the column, phase B was increased by 95% at minute 10 and kept for 4 min with a subsequent re-equilibration by decreasing phase B down to 5%. The total analysis time was 17 min. For quantification, an 11-point calibration curve was analyzed, determining the slope, intercept, and regression coefficient, and analytes concentration in the samples was calculated applying the model previously described [53]. The values were normalized to total proteins. Protein quantification was done with a BCA kit.

### PGE2 ELISA assay

PGE2 levels were measured in culture medium of A549, A427, H1264, H358, H596, H838, H125, and HCC95 human NSCLC cell lines using solid-phase sandwich enzyme-linked immunosorbent assay (ELISA). Briefly, for every cell line 3-3.5 × 10^5^ cells were plated in six-well plates in a standard volume of culture medium. The supernatant was collected at 24 h, and the assay was performed according to the manufacturer’s protocol (Cayman Chemical, 514010). The assay had a range from 7.8 to 1000 pg/ml and its sensitivity was ~15 pg/ml. Proteins from the adherent cells were then extracted in a standard volume of RIPA buffer and the total protein content was used as normalization factor for PGE2.

### Immunoblotting

Cells were lysed in RIPA buffer (50 mM Tris-HCl pH 8.0, 150 mM NaCl, 1.0% NP-40, 0.5% sodium deoxycholate, 0.1% SDS) containing complete EDTA-free protease inhibitors (Roche) and 1 mM PMSF. Samples were resolved by SDS-PAGE in Bio-Rad blotting chamber, transferred to nitrocellulose membrane using a semi-dry chamber (Bio Rad) and blocked in 5% BSA. Membranes were then incubated overnight at 4 ^o^C with primary antibody diluted in 5% BSA in TBS containing 0.1% Tween. Secondary fluorescent-tagged antibodies were from Li-Cor biosciences, and development was done in Li-Cor fluorescence–chemiluminescence detector. All antibodies and their dilutions are listed in Supplementary Table 2.

### RT-PCR

RNA was extracted using the RNAeasy kit (QIAGEN, 74104), and cDNA was synthesized with the RevertAid First Strand cDNA Synthesis Kit (Thermo Scientific, K1622). RT-PCR was performed in 96-well plates (TreffLab) with FastSybr green (Thermo Scientific, 4367659). The normalization was performed with the ΔΔCT method. The full list of the oligonucleotides used can be found in Supplementary Table 2.

### Cell proliferation assay

Cells were plated at low confluency in 24-well plates (8000 cells/well for A549, 9000 cells/well for H358, H1264, and A427) and allowed to proliferate for 48 or 96 h. Cell viability was measured by crystal violet (Sigma-Aldrich) staining (0.1% in 20% methanol) of adherent cells after 10 min fixation in 4% paraformaldehyde (Sigma-Aldrich). After washing twice and air-drying, stained cells were de-colored with 5% acetic acid, and OD_600_ was measured with a spectrophotometer.

### Soft agar colony formation assay

Cells (3 × 10^5^/well) were seeded on semi-solid agar medium (bottom layer 0.6% and top layer 0.4% mixed with cells) in a six-well plate. After 14–21 days cells were formalin-fixed and stained with 0.005% Iodonitrotetrazolium. The colonies were counted using a microscope.

### Statistical analysis

All data sets were organized and analyzed in Microsoft excel 2016 and GraphPad Prism version 7.0.0 (GraphPad Software, San Diego, CA, USA, www.graphpad.com). All data presented are expressed as mean ± SEM or ±SD of three or more biological replicates/group (except for the in vivo experiments and the human data analysis, the number is indicated in the related figure legends). The mass spectrometry-based lipidomics analyses and PGE2 measurements were repeated twice. The significance of the results was determined by employing two-tailed unpaired Student’s *t* test and one- or two-way ANOVA (Tukey’s post test) when more than two groups were compared, and significance is indicated in the related figure legends. No outliers were found in any data set and no animals or data were excluded from statistical analysis.

## Supplementary information

Supplementary figures and figure legends

Supplementary Table 1

Supplementary Table 2
