# Supplementary material for: The ACSL3-LPIAT1 signaling drives prostaglandin synthesis in non-small cell lung cancer
Source: Oncogene. 2020 Feb 7;39(14):2948–60. doi: 10.1038/s41388-020-1196-5 (PMC7118021; doi:10.1038/s41388-020-1196-5)
Supplement: Supplementary file 1 — Supplementary figures and figure legends [file 41388_2020_1196_MOESM1_ESM.docx]

**The ACSL3-LPIAT1 signaling drives prostaglandin synthesis in non-small cell lung cancer**

**Authors:** Maria Saliakoura^1^, Inés Reynoso-Moreno^2^, Chiara Pozzato^1^, Matteo Rossi Sebastiano^1^, Mirco Galié^3^, Jürg Gertsch^2^, Georgia Konstantinidou^1, 4, *^

**Affiliations:**

^1^Institute of Pharmacology, University of Bern, 3010 Bern, Switzerland. ^2^ Institute of Biochemistry and Molecular Medicine, University of Bern, 3012 Bern, Switzerland. ^3^Department of Neuroscience, Biomedicine and Movement, University of Verona, 37134 Verona, Italy. ^4^Lead contact. **^*^**Correspondence: [georgia.konstantinidou@pki.unibe.ch](mailto:georgia.konstantinidou@pki.unibe.ch)

**Running title: ACSL3-LPIAT1 drive prostaglandin synthesis**

**Supplemental Figures and figure legends**

**Figure 1. ACSL3 drives prostaglandin synthesis in NSCLC.**

**(A)** Detectable PI (mol%/sample) lipid species 72h after ACSL3 knockdown in A549 cells. Cells were transduced with either an empty vector control (pLKO) or an shRNA against ACSL3 and 72h later lipids were extracted and analyzed by mass spectrometry-based shotgun lipidomics. PC: phosphatidylcholine, PS: phosphatidylserine, PE: phosphatidylethanolamine, PI: phosphatidylinositol. n = 4/group. Data are presented as mean ± SD. Statistical analyses were done using two-tailed unpaired Student’s *t* test. **p < 0.05,* ***p < 0.01,* *** *p < 0.001,* **** *p < 0.0001*. **(B)** Immunoblot of A549 cells transduced prior performing lipidomic analysis. Cells were transduced with either an empty vector control (pLKO puro) or an shRNA against ACSL3 and 72h later lipids were extracted and analyzed by immunoblot for the indicated targets. (**C)** Relative viability of the indicated mutant KRAS cell lines 72h after transduction either with an empty vector control (pLKO puro empty) or an shRNA against ACSL3. (**D)** Immunoblot of the indicated cell lines transduced either with an empty vector control (pLKO puro empty) or an shRNA against ACSL3 and 72h later proteins were extracted and analyzed by immunoblot for the indicated targets. (**E)** Relative viability of the indicated wild-type KRAS cell lines 72h after transduction either with an empty vector control (pLKO puro empty) or an shRNA against ACSL3. (**F)** PGE2 ELISA assay for the indicated wild-type KRAS cell lines. Cells were transduced with either an empty vector control (pLKO) or shRNA against LPIAT1 (shLPIAT1 #1). Cells were transduced, selected with puromycin and plated for PGE2 measurement. 24h later the media was changed, incubated for additional 24h and PGE2 production was quantified from the supernatant using ELISA assay kit. n = 3/group. Data are presented as mean ± SD.

**Figure 2. LPIAT1 requires ACSL3-derived arachidonoyl-CoA for prostaglandin synthesis.**

**(**A) Real-time PCR for *LPIAT1* to assess knockdown efficiency of 3 different shRNAs against *LPIAT1* in the indicated mutant KRAS cell lines. Cells were transduced with 3 different shRNAs against *LPIAT1* and RNA extraction was performed 72h later followed by cDNA synthesis and real-time PCR. Data are presented as mean ± SD. (**B)** PGE2 ELISA assay for the indicated wild-type KRAS cell lines. Cells were transduced with either an empty vector control (pLKO) or shRNA against LPIAT1 (shLPIAT1 #1). Cells were transduced, selected with 2μg/mL puromycin and plated for PGE2 measurement. 24h later the media was changed, incubated for additional 24h and PGE2 production was quantified from the supernatant using ELISA assay kit. n = 3/group. Data are presented as mean ± SD. **(C**) Real-time PCR for *LPIAT1* to assess knockdown efficiency of 3 different shRNAs against *LPIAT1* in the indicated wild-type KRAS cell lines. Cells were transduced with 3 different shRNAs against *LPIAT1* and RNA extraction was performed 72h later followed by cDNA synthesis and real-time PCR. Data are presented as mean ± SD. **(D)** Relative cell number of the indicated wild-type KRAS cell lines transduced with an empty vector control (pLKO) or 3 different shRNAs against LPIAT1. n = 3/group. Data are presented as mean ± SD. **(E)** Relative cell number of H358 cell line transduced with either pLKO.1 hygro + pLenti-GIII-CMV-GFP-2A-Puro (control) or pLKO.1 hygro-shACSL3 #2 + pLenti-GIII-CMV-GFP-2A-Puro (shACSL3) or pLKO.1 hygro + pLenti-GIII-CMV-GFP-2A-Puro-LPIAT1 (LPIAT1 overexpression) or pLKO.1 hygro-shACSL3 #2 + pLenti-GIII-CMV-GFP-2A-Puro-LPIAT1 (shACSL3 + LPIAT1 overexpression). n = 3/group. Data are presented as mean ± SD. **(F)** Real-time PCR for *ACSL3* and *LPIAT1* to assess knockdown efficiency (*ACSL3*) and/or LPIAT1 overexpression (*LPIAT1*) in A549 (left) and H358 (right) cells. Cells were transduced with either pLKO.1 hygro + pLenti-GIII-CMV-GFP-2A-Puro (control) or pLKO.1 hygro-shACSL3 #2 + pLenti-GIII-CMV-GFP-2A-Puro (shACSL3) or pLKO.1 hygro + pLenti-GIII-CMV-GFP-2A-Puro-LPIAT1 (LPIAT1 overexpression) or pLKO.1 hygro-shACSL3 #2 + pLenti-GIII-CMV-GFP-2A-Puro-LPIAT1 (shACSL3 + LPIAT1 overexpression). n = 3/group. Data are presented as mean ± SD.

Statistical analyses were done using two-tailed unpaired Student’s *t* test or one-way ANOVA. **p < 0.05,* ***p < 0.01,* *** *p < 0.001,* **** *p < 0.0001*.
