# Supplementary material for: The ACSL3-LPIAT1 signaling drives prostaglandin synthesis in non-small cell lung cancer
Source: Oncogene. 2020 Feb 7;39(14):2948–60. doi: 10.1038/s41388-020-1196-5 (PMC7118021; doi:10.1038/s41388-020-1196-5)
Supplement: Supplementary file 3 — Supplementary Table 2 [file 41388_2020_1196_MOESM3_ESM.docx]

| **REAGENT or RESOURCE** | | **SOURCE** | **IDENTIFIER** |
| --- | --- | --- | --- |
| Antibodies | | | |
| Rabbit polyclonal Anti-ACSL3 (WB, 1:1000) | | Thermo Fisher | Cat#29507, **RRID:AB_2546983** |
| Mouse monoclonal Anti-ACSL4 (F-4) (WB, 1:500) | | Santa Cruz | cat#sc-454  **RRID:AB_10843105** |
| Mouse monoclonal Anti-cPLA2 (4-4B-3C) (WB, 1:1000) | | Santa Cruz | cat# sc-365230  **RRID:AB_627288** |
| Mouse monoclonal Anti-LPIAT1 (FT10) (WB, 1:1000) | | Provided by Prof. H. Arai | N/A |
| Mouse monoclonal Anti-β-actin (AC-74) (WB, 1:2000) | | Sigma-Aldrich | Cat#a-5316, **RRID:AB_476743** |
| Mouse monoclonal Anti α-tubulin (WB, 1:2000) | | Sigma-Aldrich | Cat#T6199, **RRID:AB_477583** |
| Goat Anti-mouse Red IRDye 800 CW (WB, 1:10000) | | LI-COR | Cat#926-32210, **RRID:AB_621842** |
| Goat Anti-mouse Green IRDye 680 RD (WB, 1:10000) | | LI-COR | Cat#926-68070, **RRID:AB_10956588** |
| Goat Anti-rabbit Red IRDye 800 CW (WB, 1:10000) | | LI-COR | Cat#926-32211, **RRID:AB_621843** |
| Goat Anti-rabbit Green IRDye 680 RD (WB, 1:10000) | | LI-COR | Cat#926-68071, **RRID:AB_10956166** |
| Bacterial and Virus Strains | | | |
| VVC-U of Iowa-5 Ad5CMVCre | | Viral Vector Core, University of Iowa | N/A |
| Biological Samples | | | |
| Human lung frozen tissue | | University of Bern, Institute of Pathology, Translational Research Unit | N/A |
| Chemicals, Peptides, and Recombinant Proteins | | | |
| TransIT®-293 Transfection Reagent | | Mirus Bio | Cat#MIR2705 |
| Puromycin | | Gibco | Cat#A11138-03 |
| Palmitic acid, [9,10-3H] | | Hartmann Analytic GmbH | Cat#MT845 |
| 17:0-20:4 PI(4,5)P_2_ | | Avanti Polar Lipids | Cat#LM1904 |
| 17:0-20:4 PI(4)P | | Avanti Polar Lipids | Cat#LM1901 |
| Prostaglandin E2 | | Cayman Chemical | Cat#14010 |
| Prostaglandin D2 | | Cayman Chemical | Cat# 12010 |
| Prostaglandin I2 | | Cayman Chemical | Cat# 18220 |
| Prostaglandin E2-d4 | | Cayman Chemical | Cat#10007273 |
| Critical Commercial Assays | | | |
| Prostaglandin E2 ELISA Kit | | Cayman Chemical | Cat#514010 |
| Power SYBR™ Green PCR Master Mix | | Applied Biosystems | Cat#4367659 |
| KAPA HotStart Mouse Gentotyping Kit | | Kapa Biosystems | Cat#KK7352 |
| KAPA2G Fast HotStart Genotyping Mix | | Kapa Biosystems | Cat#KK5621 |
| RNAeasy kit | | QIAGEN | Cat#74104 |
| RevertAid First Strand cDNA Synthesis Kit | | Thermo Scientific | Cat#K1622 |
| FastSybr© green | | Thermo Scientific | Cat#4367659 |
| Experimental Models: Cell Lines | | | |
| Human: A549 cell line | | Dr. John Minna (UT southwestern Medical Center) | N/A |
| Human: A427 cell line | | Dr. John Minna (UT southwestern Medical Center) | N/A |
| Human: H358 cell line | | Dr. John Minna (UT southwestern Medical Center) | N/A |
| Human: H1264 cell line | | Dr. John Minna (UT southwestern Medical Center) | N/A |
| Human: H838 cell line | | Dr. John Minna (UT southwestern Medical Center) | N/A |
| Human: H596 cell line | | Dr. John Minna (UT southwestern Medical Center) | N/A |
| Human: H125 cell line | | Dr. John Minna (UT southwestern Medical Center) | N/A |
| Human: HCC95 cell line | | Dr. John Minna (UT southwestern Medical Center) | N/A |
| Human: HEK 293T cell line | | ATCC | CRL-11268™ |
| Experimental Models: Organisms/Strains | | | |
| Mouse: *B6.129SS4-kras^tm4Tyj^/J* | | The Jackson Laboratory | JAX: 008179  **RRID:IMSR_JAX:008179** |
| Mouse: *B6.129P2-Trp53^tm1Brn^/J* | | The Jackson Laboratory | JAX: 008462  **RRID:IMSR_JAX:008462** |
| Mouse: B6;129S5-Acsl3*^Gt(OST148301)Lex^/*Orl | | The European Mouse Mutant Archive | EM:02308 |
| Mouse: NOD.Cg-Prkdc^scid^ Il2rg^tm1Wjl^/SzJ | | The Jackson Laboratory | JAX: 005557**, RRID:IMSR_JAX:005557** |
| Oligonucleotides | | | |
| shRNA targeting sequence: ACSL3 #1:  CCGGGCGGACATTGAGCGAATGTATCTCGAGATACATTCGCTCAATGTCCGCTTTTTG | Sigma-Aldrich | | TRCN0000045529 |
| shRNA targeting sequence: ACSL3 #2:  CCGGGCCTTCAAGCTGAAACGCAAACTCGAGTTTGCGTTTCAGCTTGAAGGCTTTTTG | Sigma-Aldrich | | TRCN0000045532 |
| shRNA targeting sequence: LPIAT1 #1:  CCGGCGACTGCTACAGCACAGATTTCTCGAGAAATCTGTGCTGTAGCAGTCGTTTTTG | Sigma-Aldrich | | TRCN0000290095 |
| shRNA targeting sequence: LPIAT1 #2:  CCGGCCACACTTTGCATTCTCTGGTCTCGAGACCAGAGAATGCAAAGTGTGGTTTTTG | Sigma-Aldrich | | TRCN0000290096 |
| shRNA targeting sequence: LPIAT1 #3:  CCGGGCTGGCGCAGTATATCTACAACTCGAGTTGTAGATATACTGCGCCAGCTTTTTG | Sigma-Aldrich | | TRCN0000290097 |
| Genotyping Primer: 22907, *Kras* Wild type Forward: TGTCTTTCCCCAGCACAGT | Sigma-Aldrich | | N/A |
| Genotyping Primer: 22908, *Kras* common: CTGCATAGTACGCTATACCCTGT | Sigma-Aldrich | | N/A |
| Genotyping Primer: oIMR9592, *Kras* Mutant Forward: GCAGGTCGAGGGACCTAATA | Sigma-Aldrich | | N/A |
| Genotyping Primer: oIMR8543, *p53* Forward: GGTTAAACCCAGCTTGACCA | Sigma-Aldrich | | N/A |
| Genotyping Primer: oIMR8544, *p53* Reverse: GGAGGCAGAGACAGTTGGAG | Sigma-Aldrich | | N/A |
| Genotyping Primer: *Acsl3* Forward:  CTGGAACTTTGTAGACCAGGTTGG | Sigma-Aldrich | | N/A |
| Genotyping Primer: *Acsl3* Reverse:  TGGCTACTCGGGAAACTTAGGC | Sigma-Aldrich | | N/A |
| Genotyping Primer: *Acsl3* LTR2:  AAATGGCGTTACTTAAGCTAGCTTGC | Sigma-Aldrich | | N/A |
| RT- PCR Primer: Human *LPIAT1* Forward: AGCAGCTCAGACCATGTC | Sigma-Aldrich | | N/A |
| RT- PCR Primer: Human *LPIAT1* Reverse: CTGCTCCCCATCTCTTCAGC | Sigma-Aldrich | | N/A |
| RT- PCR Primer: Human *GAPDH* Forward: CAAGGTCATCCATGACAACTTT | Sigma-Aldrich | | N/A |
| RT- PCR Primer: Human *GAPDH* Reverse: GTCCACCACCCTGTTGCTGTAG | Sigma-Aldrich | | N/A |
| Recombinant DNA | | | |
| pLKO.1 puro: GACTATCATATGCTTACCG | |  | Addgene Plasmid #8453  **RRID:Addgene_8453** |
| pLKO.1 hygro: GACTATCATATGCTTACCG | | Unpublished | Addgene plasmid #24150; **RRID:Addgene_24150** |
| pLenti-GIII-CMV-GFP-2A-Puro | | Applied Biological Materials Inc. | Generated in this manuscript by eliminating LPIAT1 from the pLenti-GIII-CMV-GFP-2A-Puro-LPIAT1 |
| pLenti-GIII-CMV-GFP-2A-Puro-LPIAT1 | | Applied Biological Materials Inc. | Cat#LV215777 |
| [pCMV-VSV-G](https://www.addgene.org/8454/):  TAATACGACTCACTATAGGG | |  | Addgene Plasmid #8454  **RRID:Addgene_8454** |
| pCMV-dR8.2 dvpr: CGCAAATGGGCGGTAGGCGTG | |  | Addgene Plasmid #8455  **RRID:Addgene_8455** |
| Software and Algorithms | | | |
| GraphPad Prism v.7 | |  | https://www.graphpad.com/scientific-software/prism/ |
| GUI Xenabrowser | |  | https://xenabrowser.net |
